# Hormonally active phytochemicals and vertebrate evolution

**DOI:** 10.1111/eva.12469

**Published:** 2017-03-23

**Authors:** Max R. Lambert, Thea M. Edwards

**Affiliations:** ^1^ School of Forestry and Environmental StudiesYale UniversityNew HavenCTUSA; ^2^ Department of BiologyUniversity of the SouthSewaneeTNUSA

**Keywords:** coumestin, eco‐evolutionary dynamics, flavonoid, isoflavone, local adaptation, phytoandrogen, phytoestrogen, steroid

## Abstract

Living plants produce a diversity of chemicals that share structural and functional properties with vertebrate hormones. Wildlife species interact with these chemicals either through consumption of plant materials or aquatic exposure. Accumulating evidence shows that exposure to these hormonally active phytochemicals (HAPs) often has consequences for behavior, physiology, and fecundity. These fitness effects suggest there is potential for an evolutionary response by vertebrates to HAPs. Here, we explore the toxicological HAP–vertebrate relationship in an evolutionary framework and discuss the potential for vertebrates to adapt to or even co‐opt the effects of plant‐derived chemicals that influence fitness. We lay out several hypotheses about HAPs and provide a path forward to test whether plant‐derived chemicals influence vertebrate reproduction and evolution. Studies of phytochemicals with direct impacts on vertebrate reproduction provide an obvious and compelling system for studying evolutionary toxicology. Furthermore, an understanding of whether animal populations evolve in response to HAPs could provide insightful context for the study of rapid evolution and how animals cope with chemical agents in the environment.

## 1 Introduction

Beginning in the 1940s, there have been numerous reports from Australia that grazing sheep on fields of subterranean and red clover (*Trifolium subterraneum and T. pretense*) leads to “clover disease,” a condition of infertility that can cause lambing rates to drop by 60%–80% (Adams, 1995; Bennetts, 1944; Biggers & Curnow, 1954; Croker, Nichols, Barbetti, & Adams, 2005). Clover disease is attributed to the consumption of hormonally active phytochemicals (HAPs), particularly phytoestrogens present in clover forage (e.g., formononetin, coumestrol, genistein, and biochanin A). A ewe affected by clover disease can develop mammary gland hypertrophy, infertility, cervical deformities preventing conception, a prolapsed uterus (the uterus falls out through the vulva), or difficulty lambing. Such dramatic results have motivated development of low‐HAP clover varieties in Australia. In the United Kingdom, farmers are encouraged to avoid pasturing cattle and sheep on red clover or other legumes before and during mating to prevent clover disease (Marley, McCalman, Buckingham, Downes, & Abberton, 2011). However, Marley et al. (2011) note that more specific recommendations are not yet possible due to inadequate understanding of HAP biology. According to trade publications intended for farmers, clover disease has always been rare in the United States (U.S.), in part because subterranean clover is not used in the U.S. and because animals may be fed a broader diet that includes clover in lower proportions (Hudson, 2013; Kintzel, 2013).

**Figure 1:**
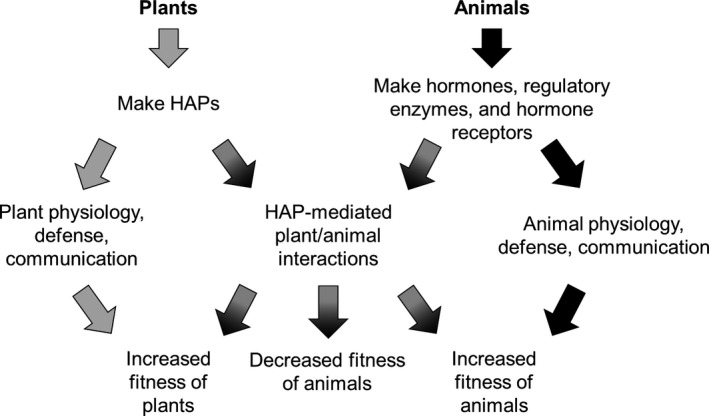
Conceptual framework illustrating the range of hypotheses explaining evolution of HAPs, influence of HAPs on animal physiology, and fitness outcomes for both plants and animals

The need for better understanding of HAP biology inspired by clover disease has since developed into a broad toxicological research field focused on how HAPs influence reproduction in a diversity of vertebrates (Rochester & Millam, 2009; Wasserman, Milton, & Chapman, 2013). Several frameworks have emerged for conceptualizing the influence of HAPs on vertebrate reproduction (Figure 1). These hypotheses primarily focus on the role of HAPs on either plant or animal fitness. Despite different interpretations of HAP effects on vertebrates, the dominant research theme frames HAPs as harmful toxins that impair animal reproduction. How environmental context (e.g., season, drought) shapes HAP production and composition and concomitant effects on vertebrates is rarely considered. Additionally, little research attention has focused on the evolutionary consequences or adaptive potential of vertebrate HAP exposure. This is surprising because environmental influences on reproduction could reasonably affect fitness and therefore evolutionary outcomes. Here, we discuss how HAPs, modulating reproductive success, might drive evolutionary change in vertebrates.

Historically, HAP research has focused on plant chemicals that elicit an estrogenic response (i.e., phytoestrogens) in vertebrates. However, we will consider HAPs to more broadly include plant chemicals and mixtures with agonistic or antagonistic effects on a range of endocrine outcomes including lipid metabolism, steroid or thyroid hormones, prolactin, or luteinizing hormone (Bovee, Schoonen, Hamers, Bento, & Peijnenburg, 2008; Chen & Chang, 2007; Higham, Ross, Warren, Heistermann, & MacLarnon, 2007; Ji, Domanski, Skirrow, & Helbin, 2007; Markiewicz, Garey, Adlercreutz, & Gurpide, 1993; Thompson, Wilson, Gobbo, Muller, & Pusey, 2008; Wang et al., 2015). Our review is representative of the hormonal pathways discussed in the literature, which is currently dominated by estrogenic compounds. We will explore how HAPs might fit into an evolutionary framework for vertebrates and discuss what research is needed to understand whether HAPs could act as a selective pressure for wildlife.

## 2 HAPs: What and Where they are

With some exceptions, most identified HAPs are flavonoids, lignins and lignans, coumestans, or saponins. There are over 9,000 distinct flavonoid and lignan structures produced via the general phenylpropanoid pathway (Ferrer, Austin, Stewart, & Noel, 2008; Winkel‐Shirley, 2001). Of these, about 100 are known to be HAPs, including genistein, daidzein, formononetin, luteolin, quercetin, resveratrol, anthocyanins, lignins and lignans, and coumestrol. Similarly, numerous saponins are produced from squalene and cholesterol via the mevalonate pathway (Faizal & Geelen, 2013; Moses, Papadopoulou, & Osbourn, 2014).

Both flavonoids and saponins are widely distributed across taxa. Flavonoids have been isolated from algae (Yoshie‐Stark, Hsieh, & Suzuki, 2003); cyanobacteria and diatoms (Scholz & Liebezeit, 2012); fungi (Qiu, Xie, Shi, Zhang, & Chen, 2010); and a myriad of land plants, including liverworts (Markham & Porter, 1979), mosses (Brinkmeier, Hahn, Seeger, Geiger, & Zinsmeister, 1999), ferns (Swain, 1980), horsetails and Ginkgo (Pietta et al., 1991), other gymnosperms (Krauze‐Baranowska, Baczek, Glod, Kaliszan, & Wollenweber, 2004), and numerous angiosperms (i.e., Condrat, Crisan, Szabo, Chambree, & Lupea, 2009). Angiosperm families with species that contain endocrine active flavonoids include Amaranthaceae, Amaryllidaceae, Brassicaceae, Cannabaceae, Dioscoreaceae, Fabaceae, Lamiaceae, Moraceae, Myrtaceae, Rosaceae, Theaceae, and Vitaceae (Bacciottini et al., 2007; Mikšátková, Lanková, Huml, & Lapčík, 2014; Wasserman, Chapman, et al., 2012; Wasserman, Taylor‐Gutt, et al., 2012). Saponins are also broadly distributed, with medically relevant saponins being found in members of at least 53 plant families (Sparg, Light, & van Staden, 2004) as well as endophytic fungi (Wu, Yang, You, & Li, 2013).

Plant part, life stage, and environment all affect HAP content, distribution, and quantities in plants at any given time (Du, Yue, & Tian, 2012). For example, in soybean plants, HAP content is low in stems, pods, flowers, and seeds, moderate in leaves, and very high in roots, with absolute amounts changing throughout ontogeny (Morgan, Dillaway, & Edwards, 2014). Because HAPs often regulate protective chemical strategies in plants, they accumulate in plant tissues in response to stressors such as light intensity, herbivory, pathogens, metals, competition, or extreme soil moisture or temperature (Chaves, Sosa, & Escudero, 2001; Deng et al., 2012; Ferrer et al., 2008; Harborne & Williams, 2000; Iriti & Faoro, 2009; Lozovaya et al., 2005; Skadhauge, Thomsen, & von Wettstein, 1997; Wang et al., 2012; Weston & Mathesius, 2013; Winkel‐Shirley, 2002). In fact, environmentally induced HAP production is a major cause of variation across individual plants, even within the same population (Romani et al., 2003).

In addition to the above HAPs, plants produce several compounds typically thought of as vertebrate sex steroids, their metabolites, and the enzymes necessary to synthesize them (Hewitt, Hillman, & Knights, 1980; Janeczko & Skoczowski, 2005; Simersky, Novak, Morris, Pouzar, & Strnad, 2009). Estradiol and estrone have been detected in seeds or pollen of apples, date and doum palm, plums, and pomegranates (Amin, Awad, El Samad, & Iskander, 1969; Amin & Paleologou, 1973; Awad, 1974; Bennett, Ko, & Heftmann, 1966; Gawienowski & Gibbs, 1969; Heftmann, Ko, & Bennett, 1966) as well as quaking aspen catkins (Khaleel, Dillman, & Gretch, 2003), common beans (Kopcewicz, 1971), moghat roots (Amin et al., 1969), and waxyleaf nightshade (Milanesi, Monje, & Boland, 2001). Similarly, progesterone was found in extracts of loblolly pine, common foxglove, tobacco, and elecampane (Carson, Jenkins, Wilson, Howell, & Moore, 2008; Simersky et al., 2009); 17‐alpha‐hydroxyprogesterone and 16‐dehydro‐progesterone occurred at significant concentrations in foxglove*;* and androstenedione was found in tobacco and elecampane (Simersky et al., 2009). Leaves and flowers of chaste trees contain progesterone, 17‐alpha‐hydroxyprogesterone, testosterone, epitestosterone, and androstenedione (Sadenkrehula, Kustrak, & Blazevic,^1991^). In aspen, estradiol content is correlated with flower maturation, suggesting that this estrogen has important, conserved reproductive functions in plants as well as animals (Khaleel et al., 2003). In fact, the surprising presence of these steroids in plants suggests that some vertebrate hormones might be more generally classified as eukaryotic hormones.

## 3 Physiological and Ecological Function of HAPs in Plants

Given the wide distribution and diversity of HAPs, it follows that HAPs are both evolutionarily old and also support a wide array of plant functions (Buer, Imin, & Djordjevic, 2010). It is likely that products of phenylpropanoid biosynthesis were critical in the evolution of land plants: Lignins provide structural support for terrestrial plants, and some flavonoids are UV protective (Ferrer et al., 2008; Pollastri & Tattini, 2011; Tossi, Lombardo, Cassia, & Lamattina, 2012; Yoo, Lee, & Patil, 2013). Both saponins and flavonoids function in allelopathy and plant defense against foreign organisms (Biate et al., 2014; Faizal & Geelen, 2013; Iriti & Faoro, 2009; Weston & Mathesius, 2013); they regulate seed dormancy and germination, root growth and gravitropism, movement of auxin, and root nodulation (Brown et al., 2001; Buer & Muday, 2004; Carlsen, Understrup, Fomsgaard, Mortensen, & Ravnskov, 2008; Faizal & Geelen, 2013; Jia et al., 2012; Nair, Safir, & Siqueira, 1991; Peer, Blakeslee, Yang, & Murphy, 2011; Peters, Frost, & Long, 1986). In fact, isoflavonoids involved in recruitment of nitrogen fixing bacteria and root nodulation can account for 9% of the dry weight of red clover (Dornstauder et al., 2001). This high isoflavonoid content no doubt contributes to clover's ability to disturb sheep fertility. As noted by Morgan et al. (2014), HAP content in plants tends to be highest in roots, which come in contact with a wide range of soil organisms, some symbiotic and some pathogenic (Balmer, Villacres de Papajewski, Planchamp, Glauser, & Mauch‐Mani, 2013). Presumably plants employ different phytochemicals to accomplish these contradictory tasks of nurturing some microbes while deterring others. Coevolution of plants and their symbionts might also select for symbionts that tolerate antipathogen phytochemicals. Finally, many flavonoids contribute to pollen fertility and serve as pigments in flowers, fruits, and seeds (Harborne & Williams, 2000; Thompson et al., 2008; Winkel‐Shirley, 2001). Given that HAPs play many roles in plants, it is likely these chemicals evolved originally for ecological or physiological functions in plants, rather than as a reproductive toxicant for vertebrates.

## 4 HAP Effects on Vertebrates

In the context of biodiversity, HAPs exemplify the principle, with their numerous structures and functions. More remarkable is their ability to communicate across taxonomic boundaries. Genistein, for example, recruits nitrogen fixing bacteria to legume roots (Subramanian, Stacey, & Yu, 2006) and binds vertebrate estrogen receptors that modulate reproduction, behavior, and metabolism (Casanova et al., 1999; Cederroth & Nef, 2009; Nowicka‐Stanczyk, Szkudelski, Szkudelska, & Nogowski, 2012; Patisaul & Polston, 2008; Viglietti‐Panzica, Mura, & Panzica, 2007). In frogs and rats, genistein alters thyroid hormone signaling and thyroid morphology and reduces thyroid hormone receptor transcription (Ji et al., 2007; Sosić‐Jurjević et al., 2010). In cancer models, genistein limits metastasis by inhibiting Notch‐1 and TGF‐beta signaling and promoting tumor cell apoptosis (Lee, Hwang, & Choi, 2016; Liu‐Smith & Meyskens, 2016). It is remarkable that one molecule can influence physiological function in plants and animals through such diverse mechanisms with a variety of outcomes. We hypothesize below that this convergence could be due to constraints in the anatomy of signaling molecules generally and/or shared common ancestry among signaling molecules that later radiated out to divergent taxa.

In fishes, amphibians, mammals, and birds, HAPs can change the timing, frequency, or duration of reproductive behaviors or events such as gonadal development, sexual maturation, estrous, and spawning (examples in Table 1). Additionally, HAPs can reduce gamete quality, fertilization rates, fecundity, or offspring mass and viability; alter circulating steroid hormone concentrations or gonad morphology; and feminize or masculinize sex ratios (Table 1). Although reported effects of HAPs tend toward negative influences, such as reduced egg number, there are also examples where HAPs have increased reproductive output (Rearick et al., 2014). Moreover, some studies report no effects of HAP exposure (e.g., Stevenson, Brown, Montgomery, & Clotfelter, 2011).

**Table 1:** Representative examples of fitness‐relevant effects of HAPs in vertebrates

Species	Common name	Exposure	Effects	Reference(s)
Amphibians				
* Rana catesbeiana*	American bullfrog	Genistein	Inhibition of metamorphosis	Ji et al., 2007
* Rana sylvatica*	Wood frog	Red clover (*Trifoliumpratense*) root exudates containing HAPs	Male‐biased sex ratios and accelerated metamorphic timing in males but not females	Lambert, 2015
* Rana sylvatica*	Wood frog	Mixed oak (*Quercus rubra +Q. velutina*) and red maple (*Acer rubrum*)	Oak feminized sex ratios and induced sexual size dimorphism. No maple effect	Lambert, Stoler, Smylie, Relyea, & Skelly, 2016
* Xenopus laevis*	African clawed frog	Quercetin	Feminized sex ratios and testicular morphology	Cong et al., 2006
* Xenopus laevis*	African clawed frogs	Oak (*Q. robur*) leaf leachate	Demasculinized testes: higher frequencies of testicular lacunae and testicular oogonia	Hermelink et al., 2010
Fish				
* Zoarces viviparus*	Viviparous eelpouts	Pulp and paper mill effluent, likely containing HAPs	Male‐biased sex ratios	Larsson et al., 2000; Larsson & Forlin, 2002
* Perca fluviatilis*	Perch	Pulp and paper mill effluent containing the HAP β‐sitosterol	Reduced gonad size, lower fecundity; reduced circulating estradiol and testosterone	Karels, Markkula, & Oikari, 2001
* Salmo trutta lacustris*	Lake trout	β‐sitosterol from pine pulp, found in paper mill effluent	Increased egg mortality, smaller egg size, smaller larvae	Lehtinen et al., 1999
* Gambusia holbrooki*	Eastern mosquitofish	β‐sitosterol and progesterone from loblolly pine pulp, found in paper mill effluent	Fewer embryos, lower rates of pregnancy, masculinized anal fin (gonopodium) in females, enlarged testes in males, reduction in social behavior	Toft, Baatrup, & Guillette, 2004; Orlando et al., 2007; Carson et al., 2008
* Oncorhynchus mykiss*	Rainbow trout	Genistein‐enriched diet	Accelerated testicular development, reduced sperm motility and numbers in males; delayed spawning, reduced ovulation and fertilization rates in females; lowered fry survivorship	Bennetau‐Pelissero et al., 2001
* Oreochromis niloticus*	Nile tilapia	Soybean based diet	Feminized sex ratios	El‐Sayed, Abdel‐Aziz, & Abdel‐Ghani, 2012
* Paralichthys lethostigma*	Southern flounder	Genistein‐enriched diets	Feminized sex ratios	DiMaggio, Kenter, Breton, & Berlinsky, 2014
* Ictalurus punctatus*	Channel catfish	Genistein‐enriched diets	Male‐biased sex ratios and high rates of intersex fish	Green & Kelly, 2009
* Betta splendens*	Siamese fighting fish	Genistein	No effect on testicular size, sperm concentration, or quality	Stevenson et al., 2011
* Pimephales promelas*	Adult fathead minnows	Genistein, daidzein, biochanin A, formononetin	Daidzein increased female egg production, otherwise no effect on gonad size, reproductive physiology, or secondary sex characteristics	Rearick et al., 2014
* Pimephales promelas*	Adult fathead minnows	Microbiologically degraded genistein, daidzein, and formononetin	Low egg production	Kelly et al., 2014
Mammals				
* Procolobus rufomitratus*	Red colobus monkeys	Seasonal diet heavy in young *Millettia dura* leaves containing phytoestrogens	Increased fecal estradiol and cortisol levels, increased aggression and rates of copulation, reduced time spent grooming.	Wasserman, Chapman, et al. (2012) and Wasserman, Taylor‐Gutt, et al. (2012)
* Papio hamadryas anubis*	Olive baboons	Seasonal diet of African black plum, *Vitex doniana,* which contains phytoprogestagens	Elevated progesterone metabolite levels in female fecal samples. Levels exceeded those in pregnancy, prevented sexual swelling, and reduced rates of association and copulation with males.	Higham et al., 2007
* Pan troglodytes schweinfurthii*	Chimpanzees	Seasonal diet containing fruits of *Vitex fischeri*	Dramatic increase in urinary progesterone among females.	Thompson et al., 2008
* Trachypithecusphayreicrepusculus*	Phayre's leaf monkeys	Seasonal diet of young leaves and fruit of four *Vitex* species	Elevated fecal progestins, longer cycle lengths and follicular phases, higher conception rates in wild females.	Lu et al., 2011
* Microtus pennsylvanicus*	Meadow voles	Soy phytoestrogens	Higher dietary doses increased behavioral interest in the opposite sex (proceptivity), lower doses caused equal interest in same and opposite sexes.	Pierson, Hetherington, & Ferkin, 2016
* Microtus montanus*	Montane voles	Methanolic extracts from winter wheat (*Triticumaestivum*) and salt grass (*Distichlisstricta*)	Reduced uterine weight and ovarian follicle counts.	Berger, Sanders, Gardner, & Negus, 1977
* Mus musculus*	Mice	Soy‐based diet	Reduced sperm counts and fertility	Cederroth et al., 2010
* Rattus norvegicus*	Rats	Genistein	Precocious vaginal opening and prolonged estrous cycles	Kouki et al., 2003
* Rattus norvegicus*	Rats	Resveratrol	Persistent estrous, reduced ovarian mass, reduced receptivity and copulatory behaviors	Henry & Witt, 2002
Birds				
* Junco hyemalis*	Dark‐eyed juncos	Phytoestrogens in soy	Delayed onset of reproductive physiology	Corbitt, Satre, Adamson, Cobbs, & Bentley, 2007
* Callipepla californica*	California quail	Clover containing biochanin A, genistein, formononetin	Delayed reproduction by two months, up to 80% fewer offspring	Leopold et al., 1976
* Coturnix japonica*	Japanese quail	Genistein	Reduced reproductive behaviors such as neck‐grabbing and mounting diminished development of the vasotocin system	Viglietti‐Panzica et al., 2007
* Coturnix japonica*	Japanese quail	Red clover (*Trifoliumpratense*) grown under irrigated or nonirrigated conditions	Increased oviduct mass under irrigated conditions	Rochester et al., 2009
* Coturnix japonica*	Japanese quail	Genistein	Reduced primary germ cell count	Intarapat, Sailasuta, & Satayali, 2016
* Coturnix japonica*	Japanese quail	Genistein‐enriched diets	Increased egg production, egg mass, shell thickness, and shell mass	Akdemir & Sahin, 2009
* Gallus gallus*	Chickens	Daidzein‐enriched diet after peak egg‐laying period	Increased egg‐laying rate	Ni et al., 2012
* Anas platyrhynchos*	Mallard ducks	Daidzein	Reduced egg‐laying rate and egg mass in younger ducks, but in older ducks, egg‐laying rate increased but yolk volume, hatchability, and overall fertility decreased.	Zhao et al., 2005

Not surprisingly, the effects of HAPs on vertebrate physiology, anatomy, and behavior vary depending on the context of exposure. Contextual elements include the identity of the HAP or HAPS mixture, dose, and route of exposure as well as animal species, sex, age/developmental stage, and environmental conditions (Rochester & Millam, 2009; Vajda & Norris, 2006; Wasserman et al., 2013). For example, adding daidzein to the feed of younger ducks (*Anas platyrhynchos*) decreased egg‐laying rate and egg mass, whereas in older ducks, the daidzein diet increased egg‐laying rate, although those eggs had decreased yolk volume and lower hatchability (Zhao et al., 2005).

It is worth noting that many of the abovementioned studies on how HAPs affect vertebrates make the inherent value judgment that increased fertility is positive and reduced fertility is negative. This assumption biases interpretation of results. For example, if limited food is available to feed offspring, then temporarily reduced fertility could be adaptive because the organism would save reproductive energy for more productive times. Alternatively, enhanced fertility caused by accelerated maturation, for example, could cause an organism to reproduce too early when the environment is not supportive of offspring survival. Therefore, valuation of the observed effect should be interpreted in the broader context of an animal's ecology.

### 4.1 Mechanisms of HAP effects in vertebrates

**Figure 2:**
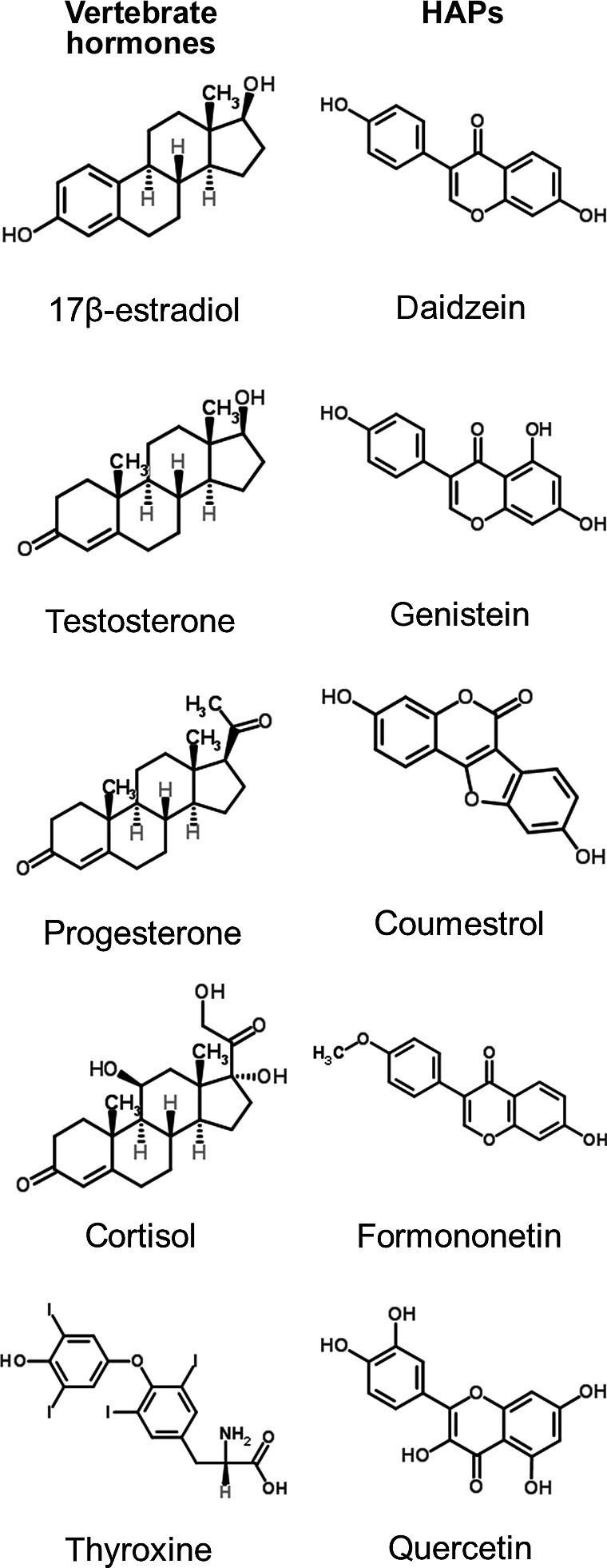
Structural examples of representative vertebrate hormones with representative hormonally active phytochemicals (HAPs). Structures provided by ChemSpider

HAPs affect animal physiology by a variety of physiological mechanisms. HAPs can bind or block animal hormones, in large part due to structural similarity (Figure 2), and thereby alter hormone‐regulated gene expression and downstream control of hormone synthesis, receptor expression, and feedback loops (Boonchird, Mahapanichkul, & Cherdshewasart, 2010; Mueller, Simon, Chae, Metzler, & Korach, 2004). Interestingly, closely related species can exhibit different levels of receptor activation by HAPs, as shown for southern white rhinoceros and one‐horned rhinoceros (Tubbs, Hartig, Cardon, Varga, & Milnes, 2012). HAPs also participate in nongenomic signaling pathways that alter phosphorylation reactions, enzymatic activity, and second messenger cascades (Greathouse et al., 2012; Lee et al., 2016). A recent survey of eleven plant species in Uganda found that extracts of leaves, bark, or flowers showed varying degrees of receptor binding in estradiol, progesterone, androgen, and cortisol assays (Wasserman et al., 2013). This survey shows that a diversity of plants and plant tissues have the potential to influence several pathways within the hypothalamic–pituitary–adrenal axis, with downstream effects on development, growth, reproduction, and behavior. We note, though, that structure and receptor binding do not necessarily confer function. Receptor binding could result in agonistic, antagonistic, or no response effects.

### 4.2 Exposure of vertebrates to HAPs

Wild vertebrates can be exposed to HAPs by consuming HAP‐containing foods or through aqueous exposure. As noted previously, HAP content in plant foods is highly variable and responsive to changing environmental conditions (reviewed by Morgan et al., 2014). There is also an apparent connection between human activity and HAP exposure among vertebrates. For example, many birds with diets high in HAPs are eating agricultural crops (Rochester & Millam, 2009). This suggests that agricultural conditions elevate HAP levels in plants, a hypothesis supported by Rochester et al. (2009) who showed that extracts of irrigated clover contained more estrogenic activity than extracts of nonirrigated clover.

In aquatic systems, there is also a connection between HAPs and human activity. HAPs, such as isoflavones, are commonly detected in agricultural waterbodies such as those near clover fields (Hoerger, Wettstein, Hungerbuhler, & Bucheli, 2009; Kolpin et al., 2010; Hoerger et al. 2011). A recent survey of contaminants in a mixed use landscape found the isoflavone daidzein in waters influenced by agriculture, golf courses, and urban wastewater (Karpuzcu et al., 2014). Similarly, work on frog ponds along a gradient of undisturbed, forested habitats to high‐density suburban neighborhoods did not detect any HAPs in forested ponds but found a diversity of HAPs (coumestrol, daidzein, formononetin, prunetin) in suburban ponds (Lambert, Giller, Barber, Fitzgerald, & Skelly, 2015) which were later shown to influence frog sex ratios (Lambert, 2015). Likewise, genistein, daidzein, and formononetin were found in two urbanized waterways but were not detected in more pristine waterways (Rearick et al., 2014). Along rivers, pulp and paper mills are associated with male‐biased sex ratios, development of male secondary sex characteristics in females, reduced gonad size, and lower fecundity in fish (Table 1, Larsson, Hallman, & Forlin, 2000; Larsson & Forlin, 2002; McMaster, Hewitt, & Parrott, 2006; Parrott, McMaster, & Hewitt, 2006). These effects are attributed to β‐sitosterol and other HAPs present in pine pulp and paper mill effluent (Table 1).

In addition to the endocrine effects of woody tissues from trees, there is emerging evidence that chemicals in foliage can act as HAPs to aquatic systems. Twenty‐four hour aqueous extracts of dead leaves from reeds, English oak, and beech show varying degrees of (anti)androgenic and (anti)estrogenic properties in yeast assays, with oak in particular showing strong estrogenic and antiandrogenic in vitro properties (Hermelink et al., 2010). When tadpoles were exposed to varying concentrations of oak leaf leachate, males had higher frequencies of testicular lacunae as well as the presence of testicular oogonia, both of which are signs of demasculinized testes (Hermelink et al., 2010).

For the most part, the detection of aqueous HAPs has been targeted to human‐dominated landscapes. Outside the two studies referenced above, no other research to our knowledge has assessed the presence of HAPs in relatively pristine environments. This is problematic as it limits inferences about the environmental contexts where vertebrate populations are exposed to HAPs. Regardless, it is clear that HAPs are commonly associated with human‐impacted environments and have the potential to influence vertebrate fitness and therefore evolution.

## 5 HAPs in a Vertebrate Evolutionary Framework

As we have shown, it is well documented that HAPs can alter vertebrate reproductive physiology, behavior, and performance. It is therefore possible that HAPs influence vertebrate fitness and natural selection, assuming there is variation in susceptibility to the effects of HAPs. Current hypotheses (sensu Wynne‐Edwards, 2001; Rochester & Millam, 2009; Wasserman et al., 2013) take different views of the evolutionary consequences of HAPs for vertebrates (Figure 1). Hughes (1988) proposed that plants make HAPs to inhibit vertebrate fertility, potentially reducing herbivory pressure. In this case, HAPs may have evolved for other physiological and ecological reasons but were repurposed to inhibit vertebrate herbivory. However, it is unclear whether reduced herbivore fecundity would minimize herbivory sufficiently in a relevant time frame for HAPs to be adaptive in this context for plants.

A second hypothesis suggests co‐evolutionary interactions between plants and vertebrate herbivores where HAP consumption may be beneficial by stimulating fertility (Wynne‐Edwards, 2001). An extension of this hypothesis is that vertebrates co‐evolved with dietary HAPs as a means to regulate reproductive status, using HAPs as indicators of environmental conditions (Berger, Negus, Sanders, & Gardner, 1981; Rochester & Millam, 2009; Wasserman et al., 2013). For example, in California quail, a temporary loss of fertility may be beneficial when the environment will not support offspring survival (Leopold, Erwin, Oh, & Browning, 1976). In this system, plants produced abundant HAPs during drought and almost no HAPs during rains. Consequently, HAPs reduced quail fertility when food was scarce but not during rains when food was plentiful. Similarly, Negus and Berger (1977) showed that nonreproductive, wintering montane voles could be stimulated into precocious reproductive activity by feeding the voles fresh green wheatgrass. The authors concluded that voles used chemical signals in the grass to “know” that spring had arrived and reproduction should begin. There is similar correlation between consumption of estrogenic *Millettia dura* leaves and induced reproductive activity in red colobus monkeys from Uganda (Wasserman, Chapman, et al., 2012; Wasserman, Taylor‐Gutt, et al., 2012).

A third hypothesis explaining interactions between HAPs and vertebrate physiology is that plants evolved HAPs to meet their own physiological and ecological needs and that HAPs affect animals by chance or due to shared ancestry in biochemical pathways. Certainly, the chemical structures of various HAPs are very similar to those of vertebrate hormones and not easily distinguished by animal hormone receptors (e.g., Bovee et al., 2008; Figure 2). Such similarities could arise due to constraints in the anatomy of signaling molecules generally. The carbon ring structure, in concert with particular side groups (such as hydroxyl groups) may be energetically or physically favored in molecular partnerships such as ligands and receptors. Aromatic rings, which are found in many hormones and HAPS, are particularly stable (Figure 2). To this end, signaling molecules may share common ancestry across taxa (Eick & Thornton, 2011).

The idea that HAPs and vertebrate hormones share a common ancestry is partially supported by several observations. First, flavonoids, estrogens, and estrogen receptors are ancient molecules (Buer et al., 2010; Pollastri & Tattini, 2011; Thornton, Need, & Crews, 2003). Flavonoids are found in algae and were likely present before the evolution of land plants (Yoshie‐Stark et al., 2003). Work by Thornton (2001) indicates that estrogens were the first steroid ligands and they probably evolved before their receptors. The first steroid receptor is thought to be a primordial estrogen receptor‐like gene that arose before the origin of bilaterally symmetric animals and then radiated out to the constellation of steroid receptors we know today (Thornton, 2001; Thornton et al., 2003). Second, Eick, Colucci, Harms, Ortlund, and Thornton (2012) report that ancient vertebrate steroid receptors recognized aromatized estrogens and evolved according to a principle of “minimal specificity” that enabled just enough variation to discriminate among endogenous steroids. In addition to minimal specificity, the ancestral binding cavity of steroid receptors was large compared to target ligands and exhibited excess hydrogen‐bonding capacity. Together, these features enable promiscuous binding of steroid receptors to a range of generally similar molecules, even if they come adorned with additional functional groups (Eick et al., 2012). The ill‐fitting nature of ancestral steroid receptors may underlie their modern‐day ability to bind both HAPs and vertebrate hormones. If modern‐day HAPs and vertebrate hormones share a common ancestor, it would explain the remarkable similarities and cross talk observed between plant and animal signaling cascades.

## 6 Testing Hypotheses about the Roles of HAPs in Vertebrate Toxicology and Evolution

Because of their direct impact on reproductive parameters, HAPs can have fitness consequences and may therefore drive evolution. The toxicological *modus operandi* is to expose laboratory strains or wild‐caught individuals from putatively naïve populations to a chemical of interest to infer whether the chemical has an effect. In published studies, HAPs have been shown to alter sex ratios, fertilization success, reproductive behaviors, gonadal development, and/or gamete quantity and quality (Table 1). While useful for understanding the toxicological effect of a particular chemical, this method limits our inference for how species may evolve in response to continued or variable exposure in the context of other environmental conditions.

More useful approaches include common garden and reciprocal transplant experiments that provide evidence that species can adapt to lethal chemicals. For example, Whitehead, Triant, Champlin, and Nacci (2010) showed that killifish (*Fundulus heteroclitus*) populations are locally adapted to polychlorinated biphenyls (PCBs; Whitehead et al., 2010). Similarly, spotted salamanders (*Ambystoma maculatum*) have become locally adapted to toxic road salt contamination (Brady, 2012), and wood frog (*Rana sylvatica*) populations are more adapted to pesticides if they live near agriculture (Cothran, Brown, & Relyea, 2013; Hua, Morehouse, & Relyea, 2012). In these studies, the populations in question have adapted to lethal chemicals, or chemicals that substantially impair development, and exert strong selective pressures. However, HAPs are interesting because they exert sublethal fitness effects by acting through reproductive pathways. To investigate adaptation to the positive and negative reproductive effects of HAPs, experimental work will need to assess reproductive endpoints rather than focusing on developmental rates or mortality. Such experiments are staples to the study of local adaptation (reviewed in Carroll, Hendry, Reznick, & Fox, 2007; Merila & Hendry, 2014) and have been widely used on studies in different contexts (e.g., Trinidadian guppies, Reznick, Bryga, & Engler, 1990; Anolis lizards in the Caribbean, Losos, 2009). As such, these well‐established methods are not unique to evolutionary toxicology but would provide useful insight into whether HAPs have influenced vertebrate evolution.

### 6.1 Mechanisms of adaptation

Populations become locally adapted when they exhibit a shift in genetically based traits that provide fitness advantages in their local environment relative to alternative environments (Kawecki & Ebert, 2004; Richardson, Urban, Bolnick, & Skelly, 2014). In particular, if natural selection acts upon standing genetic variation in HAP sensitivity, favoring individuals with lower susceptibility to HAPs and therefore higher fecundity, populations may become locally adapted to HAP ingestion or exposure. Similarly, if HAPs improve the reproductive capacity and/or outcomes such as offspring survival in a given environment, then natural selection might favor individuals that respond more to HAP exposure. Genetic variation in sensitivity to the effects of HAPs on fecundity could therefore provide an impetus for adaptive evolution.

Such genetic variation may in fact exist. Recent work shows that fish from a relatively pristine lake exhibit genetic variation in tolerance to a synthetic estrogen (17α‐ethynylestradiol) at the embryonic stage (Brazzola, Chevre., & Wedekind, 2014). While this study evaluated mortality and development of embryos, rather than reproductive parameters, it shows standing genetic variation in how individuals in a given population respond to exogenous hormonally active chemicals, indicating the potential for species to adapt to chemicals such as HAPs.

One possible mechanism for adaptation, while arguably speculative, is through changes in steroid receptor binding affinity for HAPs. Experimental work suggests that just two amino acid changes are responsible for shifting the affinity of the ancestral vertebrate estrogen receptor from estrogens to other steroids such as androgens and corticosteroids (Harms et al., 2013). This work indicates that subtle molecular changes in steroid receptor structure can have substantial effects on the receptor–ligand binding affinity as well as function. While it is unclear whether subtle evolutionary changes to the structure of steroid receptors can occur on ecologically relevant time scales and whether they can influence the affinity of these receptors for HAPs, it is possible that microevolutionary adaptation to HAPs might occur by modulating receptor–ligand interactions.

Although shifts in gene frequencies are a common sign of adaptive processes, they are not the only way that populations can secure differential reproduction and survival in response to HAPs. For example, women who consume soy isoflavones will derive greater health benefits (e.g., reduced breast cancer risk) if their intestinal microflora includes bacteria that produce favorable isoflavone metabolites (Sanchez‐Calvo, Rodriguez‐Iglesias, Molinillo, & Macias, 2013). Between 25% and 65% of the human population hosts symbiotic bacteria that alter absorption and transformation of isoflavones into metabolites with higher biological activity (Sanchez‐Calvo et al., 2013). Microflora are transmitted to offspring by contact and during vaginal delivery, making this trait transferable between generations. This example illustrates how an environmental, transgenerationally acquired trait, such as gut microbiota, can influence susceptibility to the effects of HAPs.

Similarly, environmental exposures can also cause heritable phenotypic changes by modifying the epigenome rather than the genome. In a landmark study, Anway, Cupp, Uzumcu, and Skinner (2005) showed that temporary exposure of a gestating female rat to hormonally active pesticides reduced her sons’ sperm counts, quality, and fertility; importantly, these effects continued through the succeeding four generations with no additional chemical exposure and were accompanied by heritable changes in DNA methylation patterns in the germ line. No studies to date have investigated transgenerational effects of HAPs mediated by the epigenome. But, genistein has been shown to reverse DNA hypermethylation by inhibiting DNA methyltransferase activity (Fang et al., 2005).

Of course, populations may also not be able to adapt to HAPs. It is possible that the toxicological effect of HAPs does result in reduced fitness but there is no genetic variation in susceptibility for natural selection to act upon. Maladaptive patterns may also emerge where populations exposed to HAPs have more severe fitness consequences than populations where HAPs are absent. This principle has been shown in wood frog (*Rana sylvatica*) populations impacted by road salt contamination (Brady, 2013). Specifically, wood frog larvae from ponds contaminated by road salt suffered higher rates of deformities and lower survival when experimentally exposed to road salt when compared to larvae from forested ponds with no road salt contamination.

## 7 Future Directions

HAP research would benefit from a focus on five main areas of research. First, we need to better understand where HAPs are in the environment, their quantities, degradation patterns, and under what conditions they are induced in plants. This should include studies of landscapes that are both undeveloped and anthropogenically impacted. Second, we need studies of how, when, and to what degree animals are exposed to HAPs, be it through aqueous or dietary contact. Studies that consider HAP exposure in the context of other environmental conditions (seasonality), endogenous endocrinology (e.g., estrous), and developmental stage (embryos, puberty, adulthood) would be the especially useful.

This work necessitates also understanding the agonistic and antagonistic properties of HAPs and HAP mixtures as they interact with different physiological pathways (estrogenic, androgenic, thyroid, etc.). Fully understanding the physiology of HAPs in vertebrate systems requires a third area of research, investigating the evolutionary history of hormonally active molecules (HAPs and vertebrate hormones) and their receptors. The timing of when different ligands and receptors evolved would clarify whether HAPs are adaptations or exaptations in plants.

Fourth, to assess evolutionary consequences for vertebrates, research should evaluate whether HAP exposure promotes or reduces lifetime fecundity and offspring survival. Assessing whether different HAP exposure results in fitness differences between populations is a key step for inferring fitness effects of HAPs.

The fifth area of research involves testing whether individuals within populations vary in their susceptibility to HAPs and whether different susceptibilities explain variation in fitness. Related questions would ask whether individuals from populations exposed to HAPs have higher HAP tolerances than individuals from other environments and whether populations have the capacity to adapt to HAPs or are already locally adapted. For species with long life spans, this latter step may be particularly challenging due to the logistical constraints of rearing animals from different populations to maturity or for multiple generations for common garden or reciprocal transplant experiments. However, modern molecular techniques may allow us to infer patterns of adaptation through genomic or transcriptional variations among populations (Harris, Munshi‐South, Obergfell, & O'Neill, 2013; Leionen, McCairns, O'Hara, & Merila, 2013; Munshi‐South, Zolnik, & Harris, 2016; Storz, 2005).

Powerful genomics advances have ushered us into the “omics” era where we can now understand vast variation in gene transcription (transcriptomics), protein production and structures (proteomics), and cell or tissue metabolites (metabolomics). Prior work has called for increasing genomics work in the study of hormonally active chemicals (Iguchi, Watanabe, & Yoshinao, 2006). And recent work has highlighted the fact that transcriptomics, for instance, can complement and enhance population‐level studies on the effects of hormonally active chemicals (Brander et al., 2013). HAP research can similarly benefit from increased integration of “omics” approaches.

## 8 Benefits of Studying HAPs in Toxicology and Endocrine Disruption

Toxicology traditionally investigates biological effects of anthropogenic chemicals in the environment, particularly with regard to cancer, overt birth defects, and mortality. The field of endocrine disruption has advanced classical toxicology to include more subtle effects of contaminants on health outcomes such as fertility, sexual development, metabolism, and immunity. In the course of endocrine disruption science, much has been learned about basic biology, particularly the importance of developmental processes in the establishment of dynamic lifetime physiology. For example, work in Lou Guillette's laboratory revealed new information about the role of steroidogenic enzymes in alligator temperature‐dependent sex determination while investigating the effects of estrogenic contaminants on sex reversal in alligators (Crain, Guillette, Rooney, & Pickford, 1997).

The study of how HAPs influence development and reproduction has similar benefits for understanding plant and animal physiology as well as ecological relationships between plants and animals. Perhaps more interestingly, because HAPs are effectively natural endocrine disruptors, their study may illuminate why animal endocrine systems are capable of being disrupted by contaminants. Concepts such as receptor promiscuity (the ability of a hormone receptor to bind multiple, structurally variable ligands, including ligands that are manmade) may be understood more fully in the light of evolution. There may be fitness advantages in being able to respond to diverse environmental signals, such as HAPs, which convey contextual environmental information. If HAPs increase in plant foods due to drought‐induced stress, for example, that stress might be signaled to animals through their diet and enable endocrine‐regulated acclimation to environmental change, including altered reproduction and metabolism. As global climate change progresses, HAP‐related mechanisms may play an important role in how animals respond. Because HAPs represent relatively natural interactions among plants and animals, they can provide useful evolutionary insight into broader toxicological mechanisms and responses.

## Data Archiving

There are no data associated with this manuscript to archive.
